# Impact of *KRAS*, *BRAF* and microsatellite instability status after cytoreductive surgery and HIPEC in a national cohort of colorectal peritoneal metastasis patients

**DOI:** 10.1038/s41416-021-01620-6

**Published:** 2021-12-09

**Authors:** S. G. Larsen, M. A. Goscinski, S. Dueland, S. E. Steigen, E. Hofsli, A. Torgunrud, M. Lund-Iversen, V. J. Dagenborg, K. Flatmark, H. Sorbye

**Affiliations:** 1 Section for Surgical Oncology, Norwegian Radium Hospital, Department of Gastroenterological Surgery, Oslo University Hospital, Oslo, Norway; 2 Department of Oncology, Norwegian Radium Hospital, Oslo University Hospital, Oslo, Norway; 3 Department of Clinical Pathology, University Hospital of North Norway, Tromsø, Norway; 4 The Cancer Clinic, St. Olavs Hospital, Trondheim University Hospital, Trondheim, Norway; 5 Department of Clinical and Molecular Medicine, Norwegian University of Science and Technology, Trondheim, Norway; 6 Department of Clinical Pathology, University of Oslo, Oslo, Norway; 7 Department of Tumor Biology, Institute for Cancer Research, Norwegian Radium Hospital, Oslo University Hospital, Oslo, Norway; 8 Department of Oncology, Haukeland University Hospital and Department of Clinical Science, University of Bergen, Bergen, Norway

**Keywords:** Colon cancer, Microsatellite instability

## Abstract

**Background:**

Patients with metastatic colorectal cancer (mCRC) carrying *BRAF* (mut*BRAF*) or *KRAS* mutation (mut*KRAS*) have an inferior prognosis after liver or lung surgery, whereas the prognostic role in the context of peritoneal metastasis (PM) after cytoreductive surgery (CRS) and hyperthermic intraperitoneal chemotherapy (HIPEC) has been less investigated.

**Methods:**

In total, 257 patients with non-appendiceal PM-CRC were included from the Norwegian National Unit for CRS-HIPEC.

**Results:**

In total, 180 patients received CRS**-**HIPEC with Mitomycin C, 77 patients received palliative surgery only. In the CRS-HIPEC group, mut*BRAF* was found in 24.7%, mut*KRAS* 33.9% and double wild-type 41.4% without differences in survival. MSI was found in 29.3% of mut*BRAF* cases. Patients with mut*BRAF/*MSI had superior 5-year survival compared to mut*BRAF* with MSS (58.3% vs 25.2%, *P* = 0.022), and better 3-year disease-free survival (DFS) compared to mut*KRAS* (48.6% vs 17.2%, *P* = 0.049). Peritoneal Cancer Index and the number of lymph node metastasis were prognostic for OS, and the same two, location and gender prognostic for DFS in multivariate analysis.

**Conclusions:**

PM-CRC with CRS-HIPEC patients has a surprisingly high proportion of mut*BRAF* (24.7%). Survival was similar comparing mut*BRAF*, mut*KRAS* and double wild-type cases, whereas a small subgroup with mut*BRAF* and MSI had better survival. Patients with mut*BRAF* tumours and limited PM should be considered for CRS-HIPEC.

## Introduction

Colorectal cancer (CRC) is the third most commonly diagnosed malignancy and the second leading cause of cancer death in the world [1]. Approximately 20% of patients [2, 3] have synchronous metastasis at diagnosis of CRC and 15–25% of patients develop the metachronous metastatic disease during follow-up [2, 4, 5]. The most frequent metastatic site is the liver (60–74%), whereas 19–23% of metastatic CRC (mCRC) patients have peritoneal metastases (PM) [3, 5]. PM-CRC carry a worse prognosis than isolated distant metastases at other sites [6]. Most patients with mCRC cannot be cured, illustrated by a 5-year survival of 10–20% in study patients [7, 8], and an even more grim prognosis in population-based registries with median survival 5–12 months and 5-year survival of 5–10% [9, 10].

The best chance for long-term survival for patients with mCRC is surgical resection or complete local treatment by any modality. Cytoreductive surgery followed by hyperthermic intraperitoneal chemotherapy (CRS-HIPEC) has shown promising results in patients with limited and resectable PM-CRC. Five-year survival of up to 40% has been observed in a randomised controlled trial [11], case–control studies [12–14], meta-analysis [15] and cohort studies [16]. Systemic chemotherapy alone has a limited effect on localised PM-CRC with median survival of 13–16 months [6, 17]. The aim of CRS-HIPEC is to remove all macroscopic tumours and to achieve high intraperitoneal concentrations of hyperthermic cytotoxic drugs [18]. Analysis of *BRAF*, *RAS* and microsatellite instability (MSI) status is recommended upfront in patients with mCRC to tailor systemic treatment. A potentially prognostic value of these markers could be used to aid in the selection of the most optimal patients for CRS-HIPEC. *KRAS* mutations (mut*KRAS*) occur in ~40% of patients with mCRC and is associated with a worse prognosis after liver [19] and lung surgery [20]. *BRAF* mutations (mut*BRAF*) are found in 21% of unselected population-based patients with mCRC [21], in 5–11% of trial patients [22, 23] and less in patients undergoing liver or lung metastasectomies [21, 24]. *BRAF* mutations seem to be more frequent in PM-CRC [23]. Several studies have shown a negative prognostic association of *BRAF V600E* mutations after liver or lung surgery in mCRC patients [19, 20, 23–25]. mut*BRAF* status may be a factor to consider when deciding if liver surgery should be offered in patients with very advanced mCRC [26]. MSI is present in 3–8% in patients with mCRC [27, 28]. In contrast to Stage II–III disease, MSI carries a worse prognosis in the metastatic setting [29], but is a predictive marker for the benefit of immunotherapy [30]. The relevance of MSI after surgery in mCRC is not known, but may be important as the mismatch repair system has been found important in the interpretation of *BRAF* mutations in Stage III colon cancer [31].

The possible prognostic role of *KRAS* and *BRAF* mutations has not been well studied after CRS-HIPEC in contrast to after liver or lung surgery. A recent Swiss study found that both *RAS* and *BRAF* mutations were negative prognostic factors after HIPEC [22] and a Swedish study suggested that mCRC patients with mut*BRAF* and isolated PM should rather be considered for alternative treatment options than CRS-HIPEC [32]. In a retrospective design, we studied *KRAS*, *BRAF* and MSI status in a prospective national cohort from the only national centre for CRS- HIPEC in Norway.

## Methods

### Patient population

Between January 2005 and December 2015, 335 patients with PM-CRC were considered for CRS-HIPEC at the Norwegian Radium Hospital, part of Oslo University Hospital. All patients were prospectively registered in the institutional peritoneal surface malignancy database where clinicopathological data, treatment details, and outcome were recorded. Fifty-one patients with appendiceal cancer were excluded from the study. In addition, 27 patients without histologically verified PM at primary surgery or at the time of CRS-HIPEC were excluded, resulting in a study population of 257 patients. Missing data were retrospectively collected from patient records from referring hospitals. Information regarding disease recurrence and follow-up was obtained by retrieving patient records and radiologic workup from our out-patients clinic or referring hospitals. The synchronous PM was defined as PM at or within 6 months of primary surgery and disease-free interval (DFI) was the time period from primary surgery to diagnosis of PM. The study was approved by the Norwegian Ethics Committee (s-07160b) and written informed consent was obtained from the patients.

### Treatment

CRS was performed with the intention to remove all macroscopically visible tumours, involving peritonectomy procedures and organ resections as necessary. Peritoneal tumour distribution was classified using the peritoneal cancer index (PCI) and the completeness of cytoreduction (CC) score was used to evaluate residual tumour after CRS. Complete cytoreduction (CC-0) was achieved in 180 (70%) cases and only CC-0 cases were given HIPEC. The remaining 77 cases (30%) were in a palliative setting, either because of a massive tumour load or extensive small bowel involvement. HIPEC was administrated using the open Coliseum technique until 2008, thereafter a closed technique with an open abdomen was used [33]. The HIPEC regimen contained mitomycin, 35 mg/m² (maximum 70 mg), administered for 90 min in three fractions (50% initially, 25%/30 min and 25%/60 min). Median procedure duration was 420 min (180–880) and median intraperitoneal temperature during HIPEC 42.0 °C. All anastomoses were completed before the HIPEC procedure. According to Norwegian guidelines, adjuvant chemotherapy was not routinely given. Postoperative complications (30-day morbidity and 100-day mortality) were classified according to Accordion [34].

### Histopathology and molecular analysis

Surgical specimens were collected and fixed in 4% buffered formaldehyde and subsequently embedded in paraffin followed by routine histological investigation on 3–4-µm-thin haematoxylin–eosin-stained slides. In 18 cases, tumour tissue was frozen in liquid nitrogen immediately after resection and stored at −80 °C in a tissue bank.

All cases with unavailable or unknown mutational status were retrospectively collected and reviewed by a pathologist (~100 cases). DNA eluat from previous ancillary tests was used if available. If unavailable, DNA was extracted from representative tumour areas using a Qiagen kit (Hilden, Germany). DNA quality was measured with Nanodrop^TM^ (ThermoFisher, Waltham, Massachusetts, U.S.) and analysed for mut*KRAS* in exons 2, 3 and 4 (*KRAS* Mutation Analysis, Entrogen) and for *BRAF* (V600E/K/D mutations) investigated with allele-specific real-time PCR (in-house setup, protocol available on request). *KRAS* exon 2, 3 and 4 were performed or available in all cases, and when *KRAS* was wild type we added *BRAF* testing. *NRAS* was not tested in all *KRAS* wild-type cases and left out of the study for the reason that expanding RAS analyses have had very little impact on results as shown in the Nordic 7 study [35]. MSI status was determined with PCR analysis on customised molecular MSI panels with the following markers: (BAT 26 (HMSH2 intron), BAT25 (c-KIT intron), NR24 (Zinc finger 2, 3’UTR), NR21 (SLC7A8, 5’UTR), TGF-Beta-RII (c.374-3c383), BAT 40 (1p13.1), CAT25 (CASP2,3’UTR), RCC2 (5’UTR). Changes in 3/8 markers were defined as microsatellite instable phenotype. As a control, a general microsatellite stable (MSS) DNA sample was used (in-house setup, protocol available on request). The analysis was performed from the primary tumour in all synchronous cases (98) and from primary (45) or metastatic tumours (100) in metachronous cases. Most analyses have been performed in the last 3–5 years.

In analyses regarding the CRS-HIPEC group (*n* = 174), the 57 patients with palliative or explorative operations were excluded as well as 4 patients with missing tumour blocks and 1 with unsuccessful genetic analysis. One patient was lost on follow-up. When frozen tumour tissue samples were used, they were homogenised and disrupted using TissueLyzer LT from Qiagen (Hilden, Germany). DNA was then extracted from the lysate using the AllPrep DNA/RNA/miRNA Universal Kit (Qiagen, Hilden, Germany). DNA concentrations and purity were evaluated using ThermoFisher NanoDrop spectrophotometer, and the Abs_260/280_ > 1.8 for all the samples. Targeted DNA sequencing was performed using the Ion Torrent PGM Personal Genome Machine and the Ion AmpliSeqTM Cancer Hotspot Panel v2 (ThermoFisher Scientific, Waltham, MA, USA), covering ~2800 hotspot mutations in 50 cancer-related genes. The Torrent Suite Variant Caller, with the manufacturer’s recommended settings, was used to generate single nucleotide variants and small insertions/deletions with a variant allele frequency threshold of two percent. The sequencing depth exceeded 500× for 98% of all amplicons (median depth of >4000×). Every detected mutation was manually reassessed using Integrative Genomics Viewer and functionally annotated with ANNOVAR [36], using RefSeq as the underlying gene model and information from the 1000 Genomes Project (1000genomes.org) and the Catalogue of Somatic Mutations in Cancer (cancer.sanger.ac.uk/cosmic).

### Statistical analysis

Categorical variables were described using frequencies/percentages and continuous variables were described with median/range. Associations between clinicopathological parameters and the extent of surgery were analysed using chi-squared tests (Pearson’s or linear-by-linear association). Continuous variables were analysed using Kruskal–Wallis tests. Univariate analysis was performed using the Kaplan–Meier method. Survival data were obtained from the Norwegian Cause of Death Registry and patients alive on November 1, 2017 were censored. Time from PM surgery to death or censoring date in the analyses of OS and to time of peritoneal relapse, distant metastasis, death or last follow-up in analyses of disease-free survival (DFS) were used. The log-rank test was used to compare differences in survival. Factors significant in univariate analysis for OS (mutational status, PCI, number of lymph node metastasis) and for DFS (right or left-sided tumour in addition) were further examined using the multivariable Cox proportional hazards regression model (enter), as well as age and gender. The number of variables is restricted to 5 in OS analysis and 6 in DFS analyses of the 167 cases and therefore no corrections are applied. mut*BRAF* with MSS/ mut*BRAF* with MSI/ mut*KRAS/* double wild type (double wt) were tested together in the multivariate analysis because they were mutually exclusive. Statistical analyses were conducted using SPSS software (version 25.0, SPSS Inc, IL, USA). *P* < 0.05 were considered statistically significant.

## Results

### Clinical variables and histopathology

In total, 174 patients received CRS-HIPEC (treatment group) and had a median PCI of 9, whereas the 77 patients in the palliative group had median PCI of 29. Table 1 summarises the clinicopathological characteristics of the study cohort. The palliative group differed from the treatment group regarding the following parameters: more ASA 3 patients, worse T-stage, more right-sided tumours and more specification of signet ring cells in the tumours, more synchronous disease, more systemic chemotherapy and higher PCI-index. In the treatment group, 45 patients (25.0%) had Accordion groups 3–5 complications and there was no 100-day mortality. In the palliative group, 6.5% had Accordion groups 3–5 complications and there were no deaths within 30 days.

**Table 1:** Characteristics of metastatic colorectal cancer patients with radical treatment (CRS-HIPEC, *n* = 180) or palliative/ explorative treatment (*n* = 77).

Parameter	CRS-HIPEC (*n* = 180)		Palliative treatment (*n* = 77)		*P*
	*n*	%	*n*	%	
Gender					0.095
Female	115	63.9	40	51.9	
Male	65	36.1	37	48.1	
Age, median (year, range)	59	22–77	58	20–72	0.313
T-stage					**<0.001**
T1-2	1	0.6	2	2.9	
T3	74	44.0	13	18.8	
T4	93	55.4	54	78.3	
Not reported	12		8		
N-stage					0.315
N0	51	28.7	17	24.3	
N1	60	33.7	19	27.1	
N2	67	37.6	34	48.6	
Not reported	2		0		
Number of metastatic lymph nodes, median (range)	2	0–34	3	0–28	0.125
Grade of tumour differentiation					0.615
Poorly	47	31.8	19	38.0	
Moderate	94	63.5	28	56.0	
Well	7	4.7	3	6.0	
Not reported	32		27		
Signet ring cells					**0.023**
Present	15	12.5	9	31.0	
Absent	105	87.5	20	69.0	
Not reported	60	–	48	–	
Tumour location 1					**0.017**
Right colon	76	42.2	45	58.4	
Left colon and rectum	104	57.8	32	41.6	
Tumour location 2					0.367
Colon	162	90.0	72	93.5	
Rectum	18	10.0	5	6.5	
Peritoneal metastases					
Synchronous	61	33.9	37	48.1	**0.036**
Metachronous	119	66.1	40	51.9	
Chemoterapy earlier than CRS-HIPEC					**0.002**
Yes	124	68.9	40	51.9	
No	56	31.1	37	48.1	
ASA					**0.011**
1	2	1.7	3	5.3	
2	108	92.3	43	75.4	
3	7	6.0	11	19.3	
Not reported	63		20		
CEA (median, range) (µg/L)	4	1–1820	6	1–2562	0.224
CA 19-9 (median, range) (U/L)	18.5	5–1175	32	0–764	0.232
PCI					**<0.001**
0–10	111	61.7	6	7.8	
11–20	58	32.2	7	9.1	
21–30	10	5.6	37	48.1	
>30	1	0.6	27	35.1	
PCI, median (range)	9	0–28	29	2-39	**<0.001**
Mutational status					0.530
Double wt	72	41.1	32	43.8	
mut*BRAF*	43	24.6	13	17.8	
mut*KRAS*	60	34.3	28	38.4	
Missing	5	–	4	–	
BRAF					0.430
mut*BRAF* with MSS	29	16.8	13	17.8	
mut*BRAF* with MSI	12	6.9	2	2.7	
wt*BRAF*	132	76.3	58	79.5	
Missing	7	–	4	–	
MSS/MSI					0.849
MSS	96	86.5	43	91.5	
MSI	15	13.5	4	8.5	
Not analysed	69	–	30	–	
Complications					0.064
Accordion 0–2	135	75.0	72	93.5	
Accordion 3–6	45	25.0	5	6.5	
Hospital stay (median days, range)	10	5–57	7	2–24	**<0.001**
Operation time (median minutes, range)	420	180–880	150	30–485	**<0.001**

Statistically significant *p* < 0.05 values are in bold.

### Molecular analysis

We analysed tumour tissue and performed DNA analysis for mutations in *KRAS*, *BRAF* and analyses for MSS/MSI in all patients except 8 of 257 (3.1%) where tumour tissue was not obtained. There was no significant difference in the frequency between mut*KRAS*, mut*BRAF* or double wt between CRS-HIPEC and palliative groups.

Table 2 shows mutation analyses in the 174 CRS-HIPEC patients; mut*KRAS* (*n* = 59, 33.9%), mutBRAF (*n* = 43, 24.7%) and double wt (*n* = 72, 41.4%). There were significant differences regarding primary tumour location, tumour differentiation and CEA. More mut*BRAF* were found in the right colon, whereas no mut*BRAF* rectal cancer cases were found (Table 2). There were more cases with elevated CEA values in the mut*KRAS* group (61.0%) than in mut*BRAF* (44.2 %) and double wt (28.2%, *P* < 0.001). No association was seen between PCI level and mutational status (Table 2). All cases with mut*BRAF* and half of the other cases were tested for microsatellite instability (MSI). In all, 29 mut*BRAF* patients (70.7%) were MSS and 12 mut*BRAF* patients (29.3%) MSI. In mut*BRAF* tumours, MSI were more often diagnosed in cases with synchronous PM-CRC (50% vs 17.9%, *P* = 0.047), and with poorly differentiated tumours (83.8% vs 15.4%, *P* < 0.001).

**Table 2:** Tumour mutation analysis (*KRAS*/*BRAF*) in metastatic colorectal cancer patients with cytoreductive surgery and HIPEC (CRS-HIPEC) (*n* = 174).

Parameter	mut*KRAS* (*n* = 59)		mut*BRAF* (*n* = 43)		Double wt (*n* = 72)		*P*
	*n*	%	*n*	%	*n*	%	
Gender							
Female	39	66.1	32	74.4	41	56.9	0.163
Age, median (range)	59.0	23–77	60.9	33–75	57.3	22–76	0.091
pT-stage							
T1-2	0	0	1	2.4	0	0	0.686^a^
T3	21	38.2	23	54.8	28	43.1	
T4	34	61.8	18	42.9	37	56.9	
N-stage							
N0	17	29.3	14	32.6	17	25.0	0.128^a^
N1	26	44.1	11	25.6	22	31.4	
N2	16	27.6	18	41.9	31	44.3	
Missing	0		0		2		
Grade of tumour differentiation							**0.003**
Poorly	6	12.3	16	40.0	22	40.7	
Moderate	40	81.6	21	52.5	31	57.4	
Well	3	6.1	3	7.5	1	1.9	
Not reported	10		3		18		
Signet ring cells							
Yes	1	2.7	5	15.6	9	20.0	**0.039**
No	36	97.3	27	84.4	36	80.0	
Not reported	22		11		27		
Tumour location 1							
Right colon	18	30.5	30	69.8	25	34.7	**<0.001**
Left colon and rectum	41	69.5	13	30.2	47	65.3	
Tumour location 2							
Colon	54	91.5	43	100	61	84.7	**0.012**
Rectum	5	8.5	0	0	11	15.3	
Peritoneal metastases							
Synchronous	19	32.2	11	25.6	26	36.1	0.513
Metachronous	40	67.8	32	74.4	46	63.9	
Chemoterapy earlier than CRS-HIPEC							**0.024**
Yes	33	55.9	31	72.1	56	77.8	
No	26	44.1	12	27.9	16	22.2	
CEA (µg/L)							
< 5	23	39.0	24	55.8	51	71.8	**0.001**
>5	36	61.0	19	44.2	20	28.2	
Missing					1		
PCI							
1–10	35	59.3	28	66.1	42	58.3	0.997^a^
11–20	20	33.9	12	27.9	26	36.1	
>20	4	6.8	3	7.0	4	5.6	
MSI	0	0	12	29.3	3	8.1	**<0.001**
MSS	33	100	29	70.7	34	91.9	
Missing	26		2		35		
mut*BRAF* with MSI	0	0	12	29.3	0	0	**<0.001**
mut*BRAF* with MSS	0	0	29	70.7	0	0	
wt*BRAF*	59	100	0	0	72	100	
Missing	0		2		0		
Median time from peritoneal metastasis to HIPEC (months)	3	0–43	3	0–24	3	0–53	0.889
Median time from primary cancer to HIPEC (months)	13	0–69	13	0–55	10	0–81	0.995
Median DFI (months)							
0	31	52.5	23	53.5	42	58.3	0.704
1–12	14	23.7	8	18.6	10	13.9	
>12	14	23.7	12	27.9	20	27.8	
Type of recurrence at 5 years							0.819
Local recurrence	18	30.5	13	30.2	17	23.9	
Distal metastasis	20	33.9	16	37.2	26	36.6	
Both	13	22.0	8	18.6	12	16.9	
Alive	8	13.6	6	14.0	16	22.5	

^a^Linear-by-linear association.

Statistically significant *p* < 0.05 values are in bold.

### Survival

**Fig. 1:**
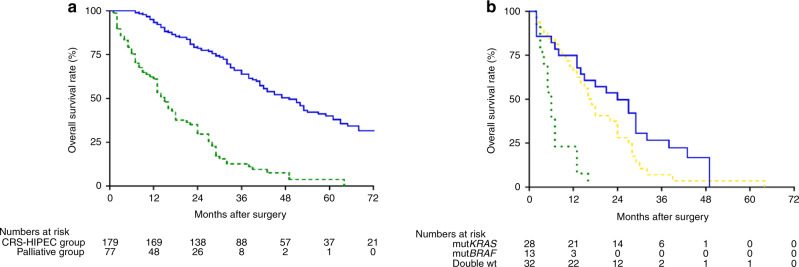
Kaplan–Meier plot showing time from surgery on the *x* axis and estimated overall survival on the *y* axis. **a** Overall survival of CRS-HIPEC versus palliative surgery. The blue line represents the CRS-HIPEC group and the green dashed line represents the patients in the palliative group. Log-rank test shows a significant difference between the two groups with *P* < 0.001. **b** Overall survival comparing mutation status after palliative resection. Kaplan–Meier plot with time from surgery on the *x* axis and estimated overall survival on the *y* axis. The blue line represents the *KRAS*-mutated tumours (mut*KRAS)* tumours and the green dotted line represents the *BRAF*-mutated (mut*BRAF)* tumours. The gold dashed line represents the patients with *KRAS* and *BRAF* wild-type (double wt) tumours. Log-rank test shows a significant difference between the three groups with *P* < 0.001.

**Fig. 2:**
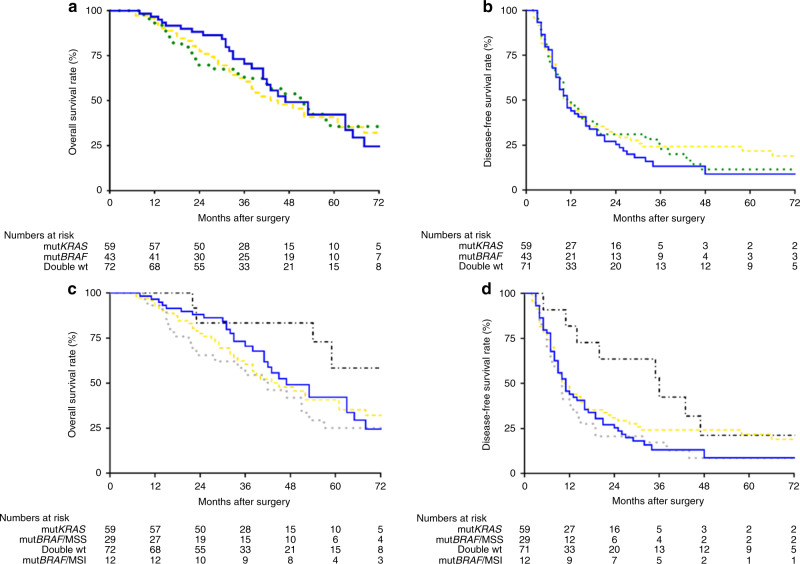
Kaplan–Meier plot with time from surgery on the *x* axis and estimated overall survival or disease free survival on the *y* axis. **a** Overall survival after CRS-HIPEC based on mutation status. The blue line represents the *KRAS*-mutated tumours (mut*KRAS)* tumours and the green dotted line represents the *BRAF*-mutated (mut*BRAF)* tumours. The gold dashed line represents the patients with *KRAS* and *BRAF* wild-type (double wt) tumours. Log-rank test shows significant difference between mut*BRAF* vs mut*KRAS*, *P* = 0.046 and between mut*BRAF* vs double wt, *P* < 0.001. **b** Disease-free survival after CRS-HIPEC based on mutation status. Kaplan–Meier plot with time from surgery on the *x* axis and estimated overall survival on the *y* axis. The blue line represents the *KRAS-*mutated tumours (mut*KRAS)* and the green dotted line represents the *BRAF*-mutated (mut*BRAF)* tumours. The gold dashed line represents the patients with *KRAS* and *BRAF* wild-type (double wt) tumours. Log-rank test is ns. **c** Overall survival comparing mutation and microsatellite instability (MSI) status. Kaplan–Meier plot with time from surgery on the *x* axis and estimated overall survival on the *y* axis. The blue line represents the *KRAS*-mutated tumours (mut*KRAS)* tumours and the green dotted line represents the *BRAF-*mutated microsatellite stable (MSS) (mut*BRAF*/MSS) tumours. The gold dashed line represents the patients with *KRAS* and *BRAF* wild-type (double wt) tumours and the black dashed/dotted line represents the *BRAF*-mutated microsatellite instable (MSI (mut*BRAF*/MSI) tumours. Log-rank test shows a significant difference between mut*BRAF* groups with MSI or MSS with *P* = 0.022. **d** Disease-free survival after CRS-HIPEC based on mutation and microsatellite instability (MSI) status. Kaplan–Meier plot with time from surgery on the *x* axis and estimated overall survival on the *y* axis. The blue line represents the *KRAS*-mutated tumours (mut*KRAS)* tumours and the grey dotted line represents the *BRAF*-mutated microsatellite stable (MSS) (mut*BRAF*/MSS) tumours. The gold dashed line represents the patients with *KRAS* and *BRAF* wild-type (double wt) tumours and the black dashed/dotted line represents the *BRAF*-mutated microsatellite instable (MSI) (mut*BRAF*/MSI) tumours. Log-rank test shows a significant difference between mut*BRAF*/MSI group and mut*KRAS* group with *P* = 0.049.

Median OS was 49 months after CRS-HIPEC in contrast to 15 months after laparotomy for the palliative group (*P* < 0.001), 5-year survival rates were 40.1% vs 3.8% (Fig. 1a and Table 3). Median DFS after CRS-HIPEC was 11 months (not shown). In the palliative group, patients with mut*BRAF* had a worse median survival (6 months) compared to patients with mut*KRAS* (24 months, *P* < 0.001) or double wt (16 months, *P* < 0.001, Fig. 1b). There was no significant difference between OS and DFS after CRS-HIPEC when stratifying for mut*KRAS*, mut*BRAF* or double wt (Fig. 2a, b). However, CRS-HIPEC patients with mut*BRAF* and MSS had shorter median OS (42 months) than those with mut*BRAF* and MSI where median survival was not reached in the study period and the corresponding 5-year OS rates were 25.2% vs 58.3% (Fig. 2c, *P* = 0.022). Patients with mut*BRAF* and MSI also had a superior DFS compared to mut*KRAS* patients (Fig. 2d, *P* = 0.049). There was no association between mutation status and type of recurrence (Table 2). PCI (HR 1.084) and the number of lymph node metastasis in the primary tumour (HR 1.056) were predictors of OS in the multivariate analysis, for every increase in PCI value or for the increase in the number of metastatic lymph nodes. Lymph node metastasis, PCI, tumour location and gender were all predictors for DFS (Table 4).

**Table 3:** Survival of metastatic colorectal cancer patients after cytoreductive surgery and HIPEC according to mutational status in univariate analysis (*n* = 174).

	All HIPEC (*n* = 179)	mut*KRAS* (*n* = 59)	mut*BRAF* total [43]	Double wt (*n* = 72)	mut*BRAF* with MSS (*n* = 29)	mut*BRAF* with MSI (*n* = 12)
Median DFS (mnt, 95% CI)	11 (9.2–12.8)	11 (7.8–14.2)	11 (7.3–14.7)	10 (7.5–12.5)	10 (8.7–11.3)	35 (11.1–58.9)
3-year DFS (%)	19.9	13.2	22.4	23.8	17.2	48.6
Median OS (mnt, 95% CI)	49 (41.7–56.3)	47 (35.2–58.8)	51 (37.7–64.3)	45 (33.3–56.7)	42 (27.1–56.9)	Not reached
5-year OS (%)	40.1	42.1	35.6	40.8	25.2	58.3

**Table 4:** Multivariable Cox regression analysis of OS and DFS after CRS-HIPEC in patients with PM-CRC (*n* = 167).

	OS			DFS		
Variable	HR	(95% CI)	*P*	HR	(95% CI)	*P*
PCI	1.084	1.05–1.12	<0.001	1.081	1.05–1.06	<0.001
Number of lymph node metastasis	1.056	1.02–1.09	0.002	1.034	1.01–1.06	0.020
Primary tumour localisation*(ref left colon/rectum)	–	–	–	0.684	0.47–0.98	0.048
Gender (ref male)	0.843	0.55–1.30	0.437	0.698	0.49–0.99	0.046
Age	0.995	0.98–1.01	0.604	1.001	0.98–1.02	0.906
Mutational status						
mut*BRAF* with MSI (ref)			0.283			0.512
mut*KRAS*	2.017	0.69–5.86	0.197	1.496	0.71–3.16	0.291
mut*BRAF* with MSS	2.83	0.94–8.52	0.064	1.591	0.72–3.50	0.249
Double wt	2.23	0.77–6.43	0.137	1.231	0.59–2.57	0.580

*OS* overall survival, *DFS* disease-free survival, *CRS* cytoreductive surgery, *HIPEC* hypertherm intraperitoneal chemotherapy, *PM* peritoneal metastasis, *CRC* colorectal cancer, *PCI* peritoneal cancer index, *MSI* microsatellite instable tumour, *MSS* microsatellite stable tumour, * tumour localisation (right colon vs left colon/rectum (ref), *ref* reference.

## Discussion

In this national cohort of PM-CRC patients treated with CRS-HIPEC, we found a high incidence of BRAF mutations (24%), and in contrast to two prior reports, we did not see any differences in survival after CRS-HIPEC according to *KRAS* or *BRAF* mutational status. Patients with mut*BRAF* and MSI had significantly better survival than all other groups. Our results suggest that mCRC patients with limited PM and mut*BRAF* should be considered for CRS-HIPEC.

### Mutations and site of metastasis

In patients with mCRC, an incidence of 35–40% mut*KRAS* and 5–20% *BRAF* mutations are usually observed [20, 21, 37]. However, the mutations seem to be associated with a distinct pattern of metastatic spread. The presence of a *KRAS* mutation is associated with a lower frequency of liver metastases and a higher frequency of lung, brain and bone metastases [38]. Patients with tumour *BRAF* mutations are less likely to present with liver limited metastasis (41% vs 63%), but these mutations are more often associated with peritoneal involvement (26% vs 14%) [23]. The mutational pattern is however different in mCRC patients receiving surgery for metastasis. In liver resected patients, mut*KRAS* are seen in 28–52% of cases [24, 39], whereas *BRAF* mutations are only in 2–5% of cases [24, 26]. In the lung, resected patients' mut*KRAS* is found in 48–62% of cases [20, 40] and BRAF mutations in 0–10% of cases [20, 40]. In mCRC patients treated with CRS-HIPEC, mut*KRAS* are reported in 42–58% of cases [19, 41]. In the far largest published study by Schneider et al. on 494 patients with CRS-HIPEC, 38% had *KRAS* mutation and only 5.8% *BRAF* mutation [22]. These results are in contrast to our results where we found a higher mut*BRAF* rate of 24.7% among our 174 CRS-HIPEC patients, and 26% by Yaeger [23]. The reason for this large difference is difficult to explain. However, *BRAF* mutations are more frequently seen in population-based cohorts compared to phase III studies and reports from tertiary referral centres [21]. The present cohort represents PM from all Norwegian patients accepted for CRS-HIPEC treatment, and thereby more accurately reflects the general population. Our results are relatively similar to Franko et al. who found 12% *BRAF* mutations in patients with multifocal mCRC including peritoneal involvement, but 18% if the patient had isolated peritoneal involvement [6].

### Mutations and CRS-HIPEC

In the evaluation of patients for resection of metastatic disease, resection of all metastatic lesions is the primary objective. However, rapid recurrence in many patients is a major challenge in the treatment of mCRC patients. Known risk factors associated with poor outcome after surgery may help to select appropriate cases for surgery. At present, the well-known factors for prognosis after CRS-HIPEC are the level of PCI [19, 42], lymph node metastasis (N + disease) [38], completeness of cytoreduction [38] and presence of signet ring cell differentiation [38–40]. In our study, PCI level (0–10 vs 10–20) and lymph node status did not vary according to mutational status, whereas signet ring cell differentiation was less frequent in cases with *KRAS* mutations.

In our study, median survival was 49 months from the time of the CRS-HIPEC and the estimated 5-year survival was 40.1% which is in concordance with results from other tertiary referral centres [15]. CRS-HIPEC is often performed some months after diagnosing PM due to recent surgery or systemic chemotherapy. When estimating survival from the first verification of PM, the median survival time for both radical and palliative treatment increases to 57 months and 20 months (*P* < 0.001) as well as the 5-year survival rates to 49.0% and 6.7%.

The use of systemic chemotherapy in CRS-HIPEC can either be given sporadically, as formal adjuvant or neoadjuvant treatment or routinely as in the PRODIGE 7 trial were nearly all cases were pretreated with six or more cycles thereby possibly selecting a population with favourable tumour biology before CRS-HIPEC. In Norway, systemic chemotherapy is routinely given in adjuvant settings to patients with N + disease, whereas neoadjuvant treatment only to selected cases with extensive PM. None of the cases in this study has received immunotherapy as this treatment was first approved in Norway in September 2019 for MSI mCRC cases.

### Mutations and survival in mCRC

In recent years, knowledge of the tumour-related genomic alterations has led to more precision-based management of patients with mCRC, both with regards to prognostic value and prediction of tumour response to systemic treatment. mut*BRAF* patients are less likely to undergo metastasectomy (26% vs 41%) [23] due to the increased risk of recurrence and worse prognosis [24–26], especially in patients with MSS tumours [43]. MSI is present in about 15% of patients with localised disease and 7% in patients with mCRC [29]. mCRC tumours with MSI are more often *BRAF* mutated compared to MSS mCRC(87% vs. 16%), and mCRC patients with MSI receive less often secondary surgery [29]. Survival rates after radical surgery for mCRC varies according to mutation status. *KRAS* mutation and especially mut*BRAF* are negative prognostic factors after liver surgery [23, 24, 44]. After hepatectomy, 5-year survival was 37% in mut*BRAF* vs 67% for wt*BRAF* [26] and median survival was inferior in mutBRAF (23 months) compared to 42 months in mut*RAS* and 63 months in double wt in another study [45]. Several authors suggest that *BRAF* status should be taken into consideration prior to liver surgery in patients with extensive liver disease [45]. However, a recent case-matched controlled study showed that mut*BRAF* is not associated with an increased risk of relapse after liver resection for mCRC, thereby supporting considering surgical treatment for resectable liver metastasis in mut*BRAF* patients [46]. Five-year survival after lung surgery in mCRC patients was 0% for mut*BRAF*, 44% for mut*KRAS* and 100% for double wt [20] with corresponding median survival rates of 15 months, 55 months and 98 months respectively. This gave rise to the question if *BRAF*-mutated patients should be excluded from lung surgery [20].

### Mutations and survival after CRS-HIPEC

Survival after CRS-HIPEC was first reported in a large study where Schneider et al found that both *KRAS* and *BRAF* mutations had a worse median cancer-specific survival: 18 months for mut*BRAF*, 38 months for mut*KRAS* compared to 52 months for double-wt patients [22]. In another study on 152 patients with CRS, results from next-generation sequencing technology were available for 68 cases: *BRAF* mutations (6.6%), but not mut*KRAS* (46.7%) were associated with worse survival [47]. Graf et al. found that *BRAF* mutations among 111 patients with PM-CRC were an independent negative prognostic marker for survival, but not *KRAS* [32]. The authors suggest that patients with *BRAF* mutations should be considered for alternative treatment options rather than CRS-HIPEC. The results from the above studies are in major contrast to our results where patients with mut*BRAF* had the same OS and DFS as mut*KRAS* and double-wt tumours. The reason for this discrepancy may be due to several factors. The study of Graf et al included appendiceal primaries and all cases considered for CRS-HIPEC including also palliative cases. This is in contrast to our results where patients receiving CRS-HIPEC had primary tumours located in the colon and rectum only, and not appendix, and in our study we also separated between HIPEC cases and palliative cases not receiving curative surgery in the end. In our palliative cases without CRS-HIPEC, BRAFmut was a poor prognostic factor. In the study of Schneider et al., only 5.8% of cases were mut*BRAF* (22/378), only 1/4th of the frequency of 24% mut*BRAF* in our study (43/180). This could be due to their function as a tertiary referral centre, which generally sees less mut*BRAF* mCRC cases than seen in the general population [21]. Data from previous publications suggest a high degree of heterogeneity in the outcome of PM-CRC patients with mut*BRAF* [48, 49]. In another study on PM-CRC patients treated with HIPEC, mut*KRAS* was not associated with survival [32].

### MSI and CRS-HIPEC

Studies of primary CRC have shown that mismatch repair status is important in their interpretation of *BRAF* mutations status, and that mut*BRAF* does not affect OS and DFS in patients with MSI tumours [50, 51]. CRC patients with MSI have less recurrence and better survival after radical surgery in Stage II–III disease [43], whereas in mCRC both MSI and mut*BRAF* are independent negative prognostic factors [52]. Sherman et al. found that patients with unresectable PM with MSI had worse survival compared to MSS PM [53]. In our study, the subgroup of the CRS-HIPEC patients with mut*BRAF* and MSI had the best survival with 5-year OS exceeding 50% and median survival not reached. Our main analysis included only PM cases treated with CRS-HIPEC, whereas Sherman et al. included all mCRC cases with PM regardless of treatment which might in part explain the difference in results [53]. Our results are supported by a study showing that liver resected mCRC patients with mut*BRAF* and MSI have a reduced risk of recurrence [46]. MSI cases are important to diagnose as 2/3 of cases benefit from immune checkpoint inhibitors (ICI) in mCRC [30]. A future research question will be how to integrate ICI in resectable mCRC MSI cases, as upfront ICI before radical surgery is promising for CRC Stage II–III with MSI [54].

### Limitations

A limitation to this study is the retrospective cohort study design, but the cohort includes all patients given CRS-HIPEC in Norway during a 11-year time period. Management of these patients has changed by utilising better preoperative staging and a shift towards using more preoperative systemic chemotherapy before CRS-HIPEC,. Patients over 75 years of age are not given CRS-HIPEC in Norway and are therefore not included in this study. The molecular data are partly obtained from the primary tumour and partly from metastatic lesions in the peritoneum. However, *RAS* and *BRAF* mutations are early molecular tumour changes, and studies have shown a good correlation between mutational status in primaries compared to metastases, and also within different metastasis in the same patient [55]. Possible heterogeneity cannot be ruled out, little data exist but this seems to be less problematic when using tissue from the primary tumour instead of metastases. MSI analyses were only partly available in cases without mut*BRAF* and analyses of *NRAS* is lacking.

### Conclusion

The study involves a large cohort of patients with PM-CRC receiving CRS-HIPEC from the Norwegian National Unit for CRS-HIPEC. A surprisingly high proportion of these patients had mut*BRAF* (24.7%). Survival after CRS-HIPEC was similar comparing mut*BRAF*, mut*KRAS* and double wt. The small subgroup with mut*BRAF* and MSI had better survival. mCRC patients with a mut*BRAF* tumour and only limited peritoneal metastasis should be considered for CRS-HIPEC.

## Supplementary information

Reproducibility checklist

## Data Availability

The datasets generated during this study are not publicly available but available from the corresponding author on reasonable request.
